# Carbohydrate dynamics in plant–fungal–nematode networks: implications for stress resilience

**DOI:** 10.3389/fpls.2026.1825021

**Published:** 2026-04-22

**Authors:** Chris A. Bell, Aishat A. Anifowose, Krzysztof Wieczorek

**Affiliations:** 1 School of Biology, Faculty of Biological Sciences, University of Leeds, Leeds, United Kingdom; 2 Department of Crop Protection, University of Ilorin, Ilorin, Nigeria; 3 Department of Agricultural Sciences, Institute of Plant Protection, BOKU University, Tulln an der Donau, Austria

**Keywords:** arbuscular mycorrhizal fungi, mutualism, plant parasitic nematode, resistance, susceptibility

## Abstract

Plant-parasitic nematodes are among the most destructive agricultural pests, driving substantial yield losses across diverse cropping systems through their exploitation of plant resources. Conversely, root-associated obligate mutualistic fungi, including arbuscular mycorrhizal fungi and endophytic taxa such as *Serendipita* spp., can reshape plant responses to nematode attack by increasing nutrient uptake, modulating stress responses, and influencing immune signalling. These fungi form intimate symbioses with plant roots, exchanging mineral nutrients for host-derived carbohydrates, placing carbon allocation at the centre of plant-fungal-nematode interactions. This review synthesises current understanding of the tripartite interactions between plants, mutualistic fungi, and plant-parasitic nematodes, with a particular focus on carbohydrate allocation as a shared and contested resource. We discuss how nematodes redirect host carbon flows to establish and maintain feeding sites, and how fungal mutualists alter carbohydrate partitioning, plant defence and stress physiology. Importantly, we highlight that fungal symbioses do not uniformly enhance resistance; in some cases, they may increase host susceptibility to nematodes by altering carbohydrate availability or defence prioritisation. By integrating findings across plant-fungal-nematode systems, this review identifies mechanisms that underpin resistance- versus susceptibility-enhancing outcomes and outlines key knowledge gaps. Together, these insights provide a framework to inform the strategic use of beneficial fungi to strengthen crop resilience against nematode stress.

## Introduction

Plant-parasitic nematodes (PPNs) are one of the most damaging crop pests causing an estimated annual yield loss of $150 billion in globally important crops such as, rice, tomato, cowpea, casava, potato, maize, citrus, soybean and yam (Gamalero and Glick, 2020; Palomares-Rius et al., 2021; Kantor et al., 2022, Kantor et al., 2024; Buzdar et al., 2025). They are obligate biotrophic plant parasites that utilise a characteristic, specialised structure, termed the stylet, to penetrate root cells to enable entry of their host (Chen et al., 2024). Three major parasitic strategies are commonly distinguished based on the feeding behaviour and mode of interaction with root: 1) Migratory ectoparasitic nematodes, such as *Longidorus* (needle nematodes), *Xiphinema* (dagger nematodes) and *Trichodorus* (stubby root nematodes). These species feed externally on plant tissues without entering the host (Smant et al., 2018). 2) Migratory endoparasitic nematodes, such as *Pratylenchus* (lesion nematodes), *Scutellonema* (yam nematodes), and *Radopholus* (burrowing nematodes). These species penetrate the root epidermis and feed whilst migrating through the cortex, causing extensive damage (Huang et al., 2019; Mathew and Opperman, 2020). 3) Sedentary endoparasitic nematodes, including *Meloidogyne* (root-knot nematodes), and *Globodera* and *Heterodera* (cyst nematodes). These nematodes colonise plant tissues by secreting effector molecules into selected host cells (Hewezi, 2015; Liu et al., 2022) to initiate the formation of permanent feeding sites that supply nutrients throughout parasitism (Gheysen and Mitchum, 2011; Bernard et al., 2017; Kleinert et al., 2017; Palomares-Rius et al., 2017; Khan et al., 2023). In addition to direct crop damage, some ectoparasitic nematodes, including *Xiphinema*, *Longidorus*, *Trichodorus*, and *Paratrichodorus* act as vectors of plant viruses, thereby increasing yield losses and enhancing plant susceptibility to secondary pathogen infections (Siddiqui and Aziz, 2024; Poudel and Yan, 2025).

Conventional management strategies for PPNs include the practice of crop rotation, the use of trap and cover crops, resistant crop varieties, soil solarisation, chemical nematicides, biotechnological approaches (e.g., nanomaterials, RNA interference technique and gene-editing tools), and the application of beneficial fungi (Abd-Elgawad, 2022; Yadav et al., 2022; Dutta et al., 2023). Among these, root-associated mutualistic fungi, particularly arbuscular mycorrhiza fungi (AM fungi), have emerged as a promising biocontrol agent (Schouteden et al., 2015; Farhaoui et al., 2025; Umer et al., 2025). AM fungi colonise plant roots and form extensive hyphal networks that deliver nutrients - especially insoluble phosphorus - to host root cells and, in return, receive photosynthetically derived carbohydrates from the host plant (Kumar et al., 2024). This symbiotic relationship can enhance plant resistance or tolerance to both biotic stresses, including PPN and insect pests, and abiotic stresses such as drought (Wu et al., 2025). Due to the obligate biotrophic nature of AM fungi and many root-infecting pathogens, including most PPNs, there is competition for both root space and host-derived resources. Carbohydrates are a key shared resource for both AM fungi and nematodes, and a deeper understanding of their allocation within plant roots could have major implications for improving plant stress resilience. Climate change is expected to exacerbate nematode-induced crop losses, particularly when combined with increasing human population pressure and global food demand (Dutta and Phani, 2023; Kantor et al., 2024), highlighting the urgent need for alternative and sustainable management strategies.

This review summarises current knowledge on the use of AM and other root-associated mutualistic fungi to manage PPNs and the mechanisms that may underpin fungal-induced resistance or susceptibility, with a particular emphasis on carbohydrate partitioning in plant-fungal-nematode interactions. We also discuss the implications of these interactions for crop resilience and highlight future research directions.

## Carbohydrate partitioning in plant-fungal symbioses

Carbohydrates, primarily in the form of sucrose, are translocated from source tissue in the leaves to sink tissues, such as roots, via the phloem. Root-associated mutualistic fungi, such as AM fungi, absorb a substantial fraction of these photosynthates - typically around 10-30% of the total carbon allocated to roots - depending on the plant and fungal species, their developmental stage, and environmental conditions (Figueiredo et al., 2021). Once assimilated, AM fungi utilise this carbon to support their own growth and metabolism and, in symbiotic networks known as common mycorrhizal networks (CMNs), potentially redistribute carbon across interconnected plants and soil compartments. These CMNs not only enhance nutrient acquisition for the host plant but may also contribute to sustained soil carbon sequestration, impacting broader ecosystem carbon cycling (Kleinert et al., 2017: Gavito et al., 2019; Genre et al., 2020; Salmeron-Santiago et al., 2022; Hannula and Morrien, 2022).

The allocation of carbon to AM fungi is tightly regulated by the host plant through complex signalling involving sugar transporters, hormonal signals (e.g., auxin, strigolactone), and defence-related pathways. Plants can modulate carbon allocation in response to fungal colonisation levels, nutrient availability, and environmental cues, maintaining a balance that ensures mutualistic benefits while preventing overexploitation by the fungus (Pozo and Azcón-Aguilar, 2007; Keller and Phillips, 2019). For example, high phosphorus availability can suppress carbon transfer to AM fungi, reflecting a feedback regulation that preserves plant fitness (Balzergue et al., 2013; Verlinden et al., 2022). Similarly, under abiotic stress such as drought or nutrient limitation, plants may redirect carbon to prioritise roots growth or stress responses, and carbon allocation to mutualists can also be affected by competition with other belowground organisms, such as nematodes or microbial communities (Xu et al., 2024; Bell et al., 2024). Although most of the work is seemingly explored in AM fungi, other mutualistic root-associated fungi share similar carbohydrate-sink traits coupled with nutrient-enhanced benefits, such as *Serendipita* (Opitz et al., 2024).

Understanding these regulatory mechanisms of carbon allocation in plant-fungal mutualist interactions is therefore essential not only for optimising symbiotic benefits but also for predicting outcomes in complex belowground ecosystems, where multiple organisms compete for host-derived resources.

## Impact of plant-parasitic nematodes on carbohydrate dynamics

Infection by PPNs profoundly alters host plant carbohydrate dynamics through the establishment of specialised feeding structures, extensive host transcriptional reprogramming, and manipulation of plant signalling pathways. Sedentary endo-parasitic nematodes, such as cyst nematodes (*Globodera* and *Heterodera*) and root-knot nematodes (*Meloidogyne*), induce the formation of nematode feeding sites, namely syncytia or giant cells, respectively. These structures function as metabolic sinks that divert photoassimilates from normal plant tissues toward nematode development, effectively overriding the plant’s endogenous source-sink relationships (Gheysen and Mitchum, 2011; Cabello et al., 2014). These feeding sites are characterised by intense metabolic activity and sustained import of carbohydrates, primarily sucrose, from surrounding tissues (Hofmann et al., 2007; Siddique and Grundler, 2015). Structural and anatomical changes accompany this metabolic reprogramming, including increased vascularisation and a higher density of phloem elements adjacent to feeding sites, which facilitates enhanced nutrient influx (Devaraja et al., 2018; Sacchi et al., 2021). Once delivered to the feeding site, sucrose is rapidly cleaved and metabolised, supporting nematode growth, reproduction, and long-term parasitism (Hofmann et al., 2007; Cabello et al., 2014).

At the molecular level, nematodes manipulate host carbohydrate metabolism by inducing the expression of genes involved in both carbohydrate synthesis and mobilisation. Upregulation of enzymes such as granule-bound starch synthase and invertases enables the temporary storage of carbohydrates as starch and their rapid remobilisation when required, ensuring a continuous supply of soluble sugars to the nematode (Bernard et al., 2017). This fine-tuned regulation reflects the nematode’s dependence on host carbon resources and highlights the central role of carbohydrates fluxes in successful parasitism. A range of plant carbohydrate transporters, such as SWEETs (Sugars Will Eventually be Exported Transporters) and monosaccharide transporters, are also upregulated in nematode feeding sites to sustain a regular influx of material that ultimately fuels nematode development (Zhao et al., 2018). The symplastic isolation of giant cells compared to the plasmodesmata-connected syncytia (Hoth et al., 2008) may suggest a different level of reliance of both types of nematode feeding structures on the expression of plant importers, however this is currently unknown.

Nematode-secreted effector proteins further contribute to this reprogramming by targeting key plant hormone pathways, including auxin, cytokinin, salicylic acid, and jasmonic acid signalling. These hormonal perturbations not only promote the initiation and maintenance of feeding sites but also suppress plant defence responses that would otherwise limit carbon diversion and feeding site stability (Hewezi, 2015). Collectively, these processes transform infected roots into persistent carbon sinks, often at significant cost to overall plant growth and productivity.

Importantly, the nematode-driven redirection of host carbohydrates places PPNs in direct competition with other belowground carbon sinks, including root-associated mutualistic fungi. Understanding how nematodes reshape carbohydrate allocation is therefore essential for predicting outcomes in complex belowground interactions and for developing strategies that limit parasite access to host-derived carbon.

## Fungal-mediated carbohydrate dynamics in plant-nematode interactions

Beyond supporting the fungal partner, carbon allocation within the host plant has critical implications for interactions with multiple belowground organisms, including PPNs. Recent studies indicate that nematode infection can disrupt the movement of carbohydrates from plants to their fungal symbionts, thereby reshaping source-sink relationships within roots (Bell et al., 2022a, Bell et al., 2024; Magkourilou et al., 2024; Siddiqui and Aziz, 2024; Wieczorek and Bell, 2025). Conversely, fungi-mediated carbon partitioning can strongly influence nematode development and population dynamics, either enhancing plant defence or, in some contexts, inadvertently increasing host susceptibility. These outcomes depend on the fungal species involved, host genotype, and prevailing environmental conditions.

During concurrent colonisation by root-associated mutualistic fungi and PPNs, competition for host-derived carbon occurs within the root system. The systemic reconfiguration of resource allocation in roots experiencing the resource sink induced by fungal associations often reduces carbon availability to parasitic nematodes and can, in some instances, suppress nematode establishment and reproduction (Bolouri Moghaddam and Van den Ende, 2012; Poveda et al., 2020; Chen et al., 2023; Fiorilli et al., 2024; Chaudhary et al., 2025; Mendes et al., 2025). Host plants respond to this competition through coordinated regulatory mechanisms, for example the sustained expression of genes involved in fatty acid biosynthesis and lipid transfer to AM fungi, while simultaneously downregulating certain mycorrhizal-induced hexose transporters such as SWEET and monosaccharide transporter genes (Bell et al., 2024). This shift restricts the pool of readily available simple sugars in root tissues, thereby limiting the carbon resources accessible to nematodes (Schouteden et al., 2015; Bell et al., 2022a, Bell et al., 2022b). This systemic reallocation of resources is also consistent in other fungal mutualists in addition to AM fungi, such as *Serendipita* (Opitz et al., 2024). The mechanisms regulating this are currently unknown, however, they likely involve different processes that are either actively reprogrammed by each symbiont or altered by the plant to promote the interaction. For example, promoters containing CTTC *cis-*regulatory element may drive expression of genes involved in AM symbiosome development (Lota et al., 2013).

Such fungal-mediated modulation of carbohydrate dynamics frequently results in enhanced plant resistance or tolerance to nematode infection (summarised in **Table 1**). For example, single or combined application of *Funneliformis mosseae, Rhizophagus fasciculatus*, and *R. intraradices* was shown to activate defence-related biochemical pathways in rice and reduce susceptibility to *M*. *graminicola*, with combined inoculation producing the strongest effect (Malviya et al., 2023). Similarly, eggplants inoculated with *Glomus mosseae* and *Gigaspora gigantea*, in combination with the phosphate-solubilising bacterium *Pseudomonas fluorescens*, exhibited reduced gall formation and enhanced biochemical defence responses against *M*. *javanica* (Sharma et al., 2021). Comparable resistance-inducing effects have been observed in tomato inoculated with *Scutellospora heterogama* against *M. incognita* (Sulieman et al., 2025), corroborating earlier findings (Schouteden et al., 2015; da Silva Campos, 2020; Liu et al., 2022). Further, *Arabidopsis* plants colonised by the fungal endophyte *Serendipita indica* exhibited reduced susceptibility to cyst nematode *H. schachtii* (Opitz et al., 2024).

**Table 1:** Fungal mutualists associated with reduced susceptibility of different host plants to plant-parasitic nematodes.

Crop	Fungal mutualist	Reduced susceptibility to nematodes	References
Banana	*Glomus mosseae*	*M*. *incognita*	Jaizme-Vega et al., 1997
Banana	*G. mosseae*	*Pratylenchus coffeae, Radopholus similis*	Elsen et al., 2009
Coffee	*Glomus* spp.	*Pratylenchus coffeae* (together with “helper” bacteria)	Hindersah and Asyiah, 2024
Eggplant	*G. mosseae, Gigaspora gigantea*	*M. javanica*	Sharma et al., 2021; Wang et al., 2020
Tomato	*Scutellospora heterogama*	*M. incognita*	Sulieman et al., 2025
Tomato	*Funneliformis mosseae*, *Rhizophagus irregularis*	*M. incognita*	Schouteden et al., 2015
Tomato	*R. irregularis*	*M. incognita*	Vos et al., 2013
Tomato	*R. aggregatus, F. mosseae, G. gigantea*	*M. incognita*	Alamri et al., 2022
Tomato	*R. irregularis, Claroideoglomus claroideum, Gigaspora margarita, F. mosseae*	*M. javanica*	Caccia et al., 2025
Rice	*F. mosseae, Rhizophagus fasciculatus, Rhizophagus intraradices*	*M*. *graminicola*	Malviya et al., 2023
Arabidopsis	*Serendipita indica*	*H. schachtii, M. javanica*	Opitz et al., 2024

However, plant-mutualistic fungal interactions and their resultant altered carbon dynamics do not universally enhance resistance, and several studies have documented increased nematode susceptibility following colonisation (**Table 2**). For instance, wheat plants colonised by a commercial AM fungal consortium (*Claroideoglomus entunicatum, Funneliformis coronatum, Rhizophagus irregularis* and *F. mosseae*) showed an average 82% increase in *Pratylenchus neglectus* populations (Frew et al., 2018). Similarly, maize plants colonised by diverse AM fungal species, including *Rhizophagus clarus, Claroideoglomus etunicatum, Gigaspora* spp., supported higher populations of *Pratylenchus brachyurus* (Brito et al., 2018). In *Cymbopogon citratus*, colonisation by *R. clarus* and *C. etunicatum* led to an approximately ninefold increase in population of *P. brachyurus* abundance relative to non-mycorrhizal plants (Rodrigues e Silva et al., 2021). Field studies in Mexico also reported increased *Pratylechus* spp. infection in maize roots colonised by native AM fungal communities (Alvarado-Herrejón et al., 2019). In potato, AM fungal colonisation enhanced the fitness and population density of potato cyst nematodes (Bell et al., 2022a). Further, species from the order Diversisporales tended to increase *Pratylenchus* population densities compared to those from the order Glomerales (Gough et al., 2020). Finally, the endophytic fungus *Serendipita indica* was shown to mediate carbon allocation in *Arabidopsis* plants in a manner that benefited both root-knot and cyst nematodes, which exploited the altered host physiology and attenuated plant defence responses (Opitz et al., 2024).

**Table 2:** Fungal mutualists associated with induced susceptibility of different host plants to plant-parasitic nematodes.

Crop	Fungal mutualist	Induced susceptibility to nematodes	References
Wheat	*Claroideoglomus entunicatum, Funneliformis coronatum, Rhizophagus irregularis, F. mosseae*	*Pratylenchus neglectus*	Frew et al., 2018
Maize	*Rhizophagus clarus, C. etunicatum, Gigaspora rosea, G. margarita, Scutellospora calospora, S. heterogama*	*Pratylenchus brachyurus*	Brito et al., 2018
Lemon grass *(Cymbopogon citratus)*	*Rhizophagus clarus* and *C. etunicatum*	*P. brachyurus*	Rodrigues e Silva et al., 2021
Potato	*R. irregularis; Funneliformis* sp.	*Globodera pallida*	Bell et al., 2022a
Arabidopsis	*Serendipita indica*	*Heterodera schachtii* & *M. javanica* in systemic setup	Opitz et al., 2024
Grasses	*Diversisporales* spp.	*Pratylenchus* spp.	Gough et al., 2020

Together, these contrasting outcomes of reduced (**Table 1**) and enhanced susceptibility (**Table 2**) highlight the varying influences of mutualistic fungi based on plant and pathogen genotypes. The pathogens accessibility to resources, such as carbohydrates, which may be partitioned towards the fungus (plant identity dependent) will be a central determinant of the hosts ability to restrict resource flow to the pathogen, thereby reducing its susceptibility. Misallocation or the inability to control allocation may inadvertently promote nematode success and perhaps underpins observed context-dependent shifts in outcomes (**Tables 1**, **2**). More research is needed into the ability of different plants to modulate mutualism in changing environments.

## Induced resistance and susceptibility mechanisms in plant-fungi-nematode scenarios

In plant-fungal-nematode scenarios, AM fungi frequently modulate plant defence responses, resulting either in enhanced resistance or, under certain conditions, increased susceptibility to nematode infection, as discussed above. Mycorrhizal-induced resistance or mycorrhizal-induced susceptibility (MIR/MIS) encompasses a suite of molecules and mechanisms that are systemically activated or primed following AM fungal colonisation (Dreischhoff et al., 2020; Poveda et al., 2020). These responses are largely orchestrated through the systemic acquired resistance (SAR) and induced systemic resistance (ISR) signalling pathways, which are primarily regulated by salicylic acid (SA) and jasmonic acid/ethylene (JA/ET), respectively. Crosstalk between these pathways enables plants to fine-tune defence outputs in response to multiple biotic partners, including both mutualists and parasites (Cameron et al., 2013; Bedini et al., 2018; Dreischhoff et al., 2020; Kamle et al., 2020; Poveda et al., 2020; Yu et al., 2022). Importantly, any reduction in defensive systems induced by a mutualist may be exploited by nearby parasites.

Crucially, the activation of MIR/MIS is tightly linked to carbon availability and allocation within the host. AM fungal colonisation alters sink-source relationships by redirecting photosynthates towards fungal structures, which in turn affects the carbon pools available for defence-related metabolism. This carbon reprogramming supports the synthesis of defence compounds, reinforcement of cell walls, and the rapid deployment of inducible responses upon nematode attack. Depending on the balance between fungal demand, plant nutritional status, and nematode pressure, this reallocation can result in ether antagonistic outcomes (enhanced resistance to nematodes) or synergistic outcomes in which fungal colonisation indirectly benefits the parasite (Bedini et al., 2018; Kamle et al., 2020; Yu et al., 2022). Carbon investments that impact susceptibility may also reflect more general plant responses, such as increased root branching near nematode infection sites, which may provide additional entry points for other nematodes and thereby increase overall susceptibility (Guarneri et al., 2023).

AM fungi commonly prime JA/ET-dependent ISR and, to a lesser extent, SA-dependent pathways, enabling faster and stronger defence activation following nematode invasion (Schouten, 2016; Yu et al., 2022). Unlike direct antimicrobial defences, ISR relies on improved host preparedness, which is energetically costly and therefore highly dependent on sustained carbohydrate supply. AM fungal-mediated priming thus represents a strategic investment of carbon into latent defence capacity rather than constitutive defence expression. Although we discuss AM fungi directly, parallels can likely be drawn for less-researched mutualistic fungi, such as *Serendipita*, and further work is needed to discover mutualist-wide mechanisms for induced/reduced susceptibility. It is likely that these shared mechanisms will cover a new grouped phenomenon of “mutualist-induced susceptibility” rather than “mycorrhizal-induced susceptibility” in the near future.

### Structural defences and carbon-dependent reinforcement

AM fungal-induced resistance includes a range of carbon-intensive structural modifications that physically restrict nematode penetration and migration. These include thickening of cell walls through increased lignin and callose deposition, enhanced synthesis of hydroxyproline-rich glycoproteins (HRGPs), and overall strengthening root tissues (Yadav et al., 2022; Ghorui et al., 2025; Umer et al., 2025). Such processes rely on diverted carbon skeletons from primary metabolism into the phenylpropanoid and cell wall biosynthesis pathways. Additionally, AM fungal-induced changes in root architecture, such as increased lateral root branching and thickened epidermal layers, increase tolerance to nematode feeding damage and further redistribute carbon sinks within the root system (Weng et al., 2022). Fungal hyphae themselves may also function as spatial competitors, physically impeding nematode access to root tissues (Gough et al., 2020).

### Biochemical defence, secondary metabolism, and rhizosphere effects

AM fungal colonisation also enhances biochemical defences that are strongly coupled to carbohydrate metabolism. Increased carbon flux into secondary metabolic pathways supports the production of phenolics, flavonoids, alkaloids, terpenoids, and volatile organic compounds (VOCs) with nematicidal, nematostatic or repellent activity (Kaur and Suseela, 2020; Zhao et al., 2022; Dhalaria et al., 2024). These compounds not only act directly on nematodes but also shape root exudate composition, thereby restructuring the rhizosphere microbiome. Modified exudates derived from mycorrhizal roots have been shown to repel or temporarily paralyse nematode juveniles and to promote populations of antagonistic microorganisms such as *Pseudomonas* spp. and *Trichoderma* spp., further suppressing nematode infection (Zhao et al., 2022; Ghorui et al., 2025).

At the enzymatic level, MIR is associated with elevated activity of defence-related enzymes, including chitinases, β-1,3-glucanases, phenylalanine ammonia-lyase (PAL) and antioxidant enzymes such as superoxide dismutase (SOD). The induction of these enzymes reflects a reallocation of carbon and energy toward defence metabolism and redox homeostasis, which is essential for sustaining prolonged resistance responses (Kaur and Suseela, 2020).

Recent advances also show that reactive oxygen (ROS) and reactive nitrogen species (RNS) function as central integrators of stress signalling, metabolic reprogramming, and carbon allocation. These species regulate the strength and timing of SA- and JA/ET-mediated defences, coordinate antioxidant responses, and modulate carbon flux into phenylpropanoid and lignin biosynthesis (Ali et al., 2025). Their roles in linking metabolic status with immune activation highlight an additional layer of regulation that may influence whether fungal colonisation enhances resistance or predisposes plants to susceptibility. Incorporating reactive species dynamics would provide greater mechanistic depth to our understanding of how carbon availability, defence signalling, and belowground symbioses interact to determine plant resilience.

### Fungal-induced susceptibility: defence suppression and carbon diversion

In contrast to MIR, mycorrhizal-induced susceptibility (MIS) represents a condition in which fungal mutualist colonisation may suppress or attenuate plant defences in ways that favour nematode infection. MIS arises primarily from the need to maintain symbiosis, which requires partial suppression of SA-dependent defences typically effective against biotrophic pathogens, including PPNs. This suppression is often mediated through ubiquitin-proteasome system (UPS) activity, which targets key defence regulators for degradation and shifts hormonal signalling toward JA/ET dominance (Schouteden et al., 2015; Kyndt, 2023).

From a carbon perspective, MIS can be viewed as a double cost to the plants: (i) defence pathways are downregulated, reducing metabolic investments in resistance, and (ii) AM fungi act as strong carbon sinks, drawing resources away from both defence and growth. Enhanced UPS activity further increases ATP and carbon consumption, potentially exacerbating resource limitation under high nematode pressure. Nematodes may exploit this window of reduced SA-mediated defence and increased carbohydrate availability to establish feeding sites more effectively and enhance their reproduction (Opitz et al., 2024).

The factors governing an MIR versus MIS response remain unclear and require in-depth analysis of the underlying genes, their regulatory mechanisms and promoters. It is possible that promoters of MIR genes that are not induced and that do not confer inducible resistance in MIS host species, lack AM fungal-inducible elements, such as the CTTC motif (Lota et al., 2013). Identifying the elements that govern AM fungal-inducible gene expression could provide insights into how this response is systemically mediated from colonised cells. The hierarchy that determines which sink (fungus or nematode) prevails is not yet fully understood and requires spatio-temporal analysis of both interactions. The plant’s ability to allocate resources to its defence system, rather than a single symbiont, may reflect a general response to perceived biotic threats. We hypothesise that the default response favours nourishing symbionts, which shifts to defence upon recognition of a threat.

**Figure 1:**
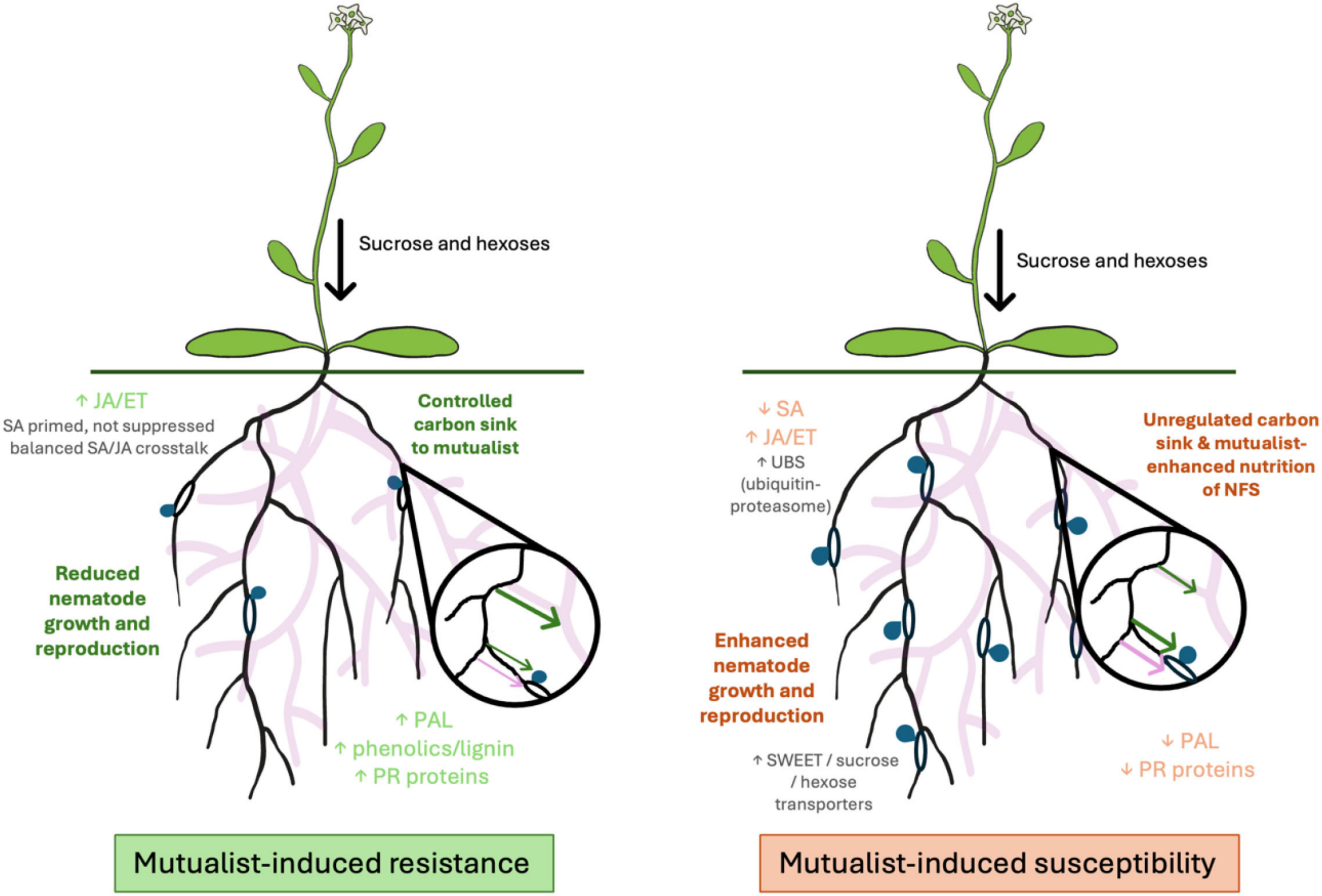
The influence of carbon flow on mutualist-induced resistance or susceptibility to plant-parasitic nematodes. Under mutualist-induced resistance (left panel), host roots (black) may preferentially allocate carbon (green arrows) to fungal symbionts (purple hyphae) and defence-related metabolism, while potentially restricting sugar availability at nematode feeding sites (NFS; nematodes coloured blue). The loss of mutualist-delivered nutrients to NFS is also restricted (purple arrow). Balanced SA–JA/ET signalling and enhanced secondary metabolite biosynthesis limit nematode development. Under mutualist-induced susceptibility (right panel), there may be unregulated losses to NFSs of photo assimilated sugars (green arrow) and mutualist-delivered nutrients (purple arrow) as a result of increased systemic transport. This may coincide with reduced carbohydrate transfer to mutualists (green arrow). Suppression of SA-dependent defences favour NFS maintenance and nematode reproduction, allowing parasites to exploit hosts with increased nutrition. In both panels the arrow thickness describes the relative amount of resource flow. The nematode illustrations depict sedentary nematodes but may be widely applicable to any plant-parasitic nematodes.

Together, MIR and MIS represent two ends of a continuum governed by hormonal crosstalk, carbon partitioning, and ecological context **Figure 1**. Whether fungal colonisation results in resistance or susceptibility depends on how effectively plants can allocate carbon to sustain mutualism-associated benefits while preventing parasitic exploitation. Understanding how carbohydrate dynamics intersect with defence signalling is therefore central to predicting and manipulating outcomes in plant-fungi-nematode systems. Multi-omics approaches are required to match inducible genes with genomic features and carbon allocation patterns to reveal how, when and where host resources are partitioned during complex biotic interactions.

## Conclusion and future perspectives

Mutualistic root-associated fungi profoundly reshape plant carbon economy, photosynthetic performance, and defence signalling, thereby positioning carbohydrates at the core of plant-fungus-nematode interactions. Enhanced carbon fixation and allocation to roots can fuel the biosynthesis of defence-related secondary metabolites, including phenolics, flavonoids, and lignins, which act both as structural barriers and as regulators of systemic immune responses mediated simultaneously by SA- and JA/ET defence pathways. In many systems, this coordinated reprogramming underpins mutualist-induced resistance, leading to reduced nematode infection and improved host tolerance. However, these benefits are highly context dependent. Increased carbon flux to colonised roots and partial suppression of SA-dependent defences required for symbiosis can, in some cases, be exploited by PPNs, resulting in mutualist-induced susceptibility. Nematodes may benefit directly from enhanced sugar availability at feeding sites or indirectly from weakened defence signalling, while parasitism itself can disrupt carbon transfer to fungal partners and attenuate their protective effects. Consequently, resistance and susceptibility should be viewed not as fixed or opposing states, but as dynamic outcomes of a shared regulatory framework governed by carbon allocation, hormonal crosstalk, and defence prioritisation. Within this continuum, plant resources are flexibly directed either toward defensive metabolism and mutualistic support or diverted to sustain parasite development **Figure 1**. These same processes that shape mutualist-induced resistance or susceptibility are also likely to contribute to host tolerance (i.e., the ability to maintain yield under pathogen pressure), and a genotype’s responsiveness to mutualist colonization may substantially influence tolerance and resistance assessments that inform agricultural decision-making (Maxwell et al., 2026).

Future progress in this field hinges on resolving how plants regulate carbon partitioning among competing belowground sinks and how this regulation intersects with defence signalling. A major challenge is to determine whether plants can be bred or engineered to preferentially allocate carbon to beneficial fungal symbionts and host defence pathways while restricting sugar flow to nematode feeding sites, without compromising symbiotic functionality or plant fitness. Addressing this question will require mechanistic insight into sugar transporter regulation, spatial and temporal carbon fluxes, and sink-source plasticity during simultaneous fungal colonisation and nematode infection.

Variation among fungal mutualists further complicates these interactions, as different species or strains can either promote resistance or inadvertently enhance susceptibility. Comparative analysis of fungal metabolic strategies, effector repertoires, and their influence on SA-JA signalling networks will be essential for identifying fungal traits associated with predictable protective outcomes. In parallel, the use of multi-species among fungal partners may generate emergent effects on carbon allocation and nematode performance.

At the molecular level, integrative multi-omics approaches combined with functional validation offer powerful tools to disentangle these complex networks. Targeted manipulation of nematode-induced host sugar transporters (e.g., SWEET, SUC, TMT, VGT), redirection of carbon toward phenylpropanoid and lignin biosynthesis, fine-tuning of JA–SA crosstalk via key transcription regulators (such as NPR1 and MYC2), and enhancement of defence-related enzymes and metabolites represent promising strategies to stabilise resistance and minimise susceptibility. Ultimately, translating these insights into practice will require predictive frameworks that integrate host phenotypes, fungal identity, nematode species, soil conditions and environmental context, supported by rigorous greenhouse and field validation. Such an integrated understanding will be critical for harnessing fungal mutualists as reliable components of sustainable, resistance-oriented crop protection strategies.
